# Comparative Study between a Novel In Vivo Method and CBCT for Assessment of Ridge Alterations after Socket Preservation—Pilot Study

**DOI:** 10.3390/ijerph16010127

**Published:** 2019-01-05

**Authors:** Vasilena Ivanova, Ivan Chenchev, Stefan Zlatev, Georgi Iordanov, Eitan Mijiritsky

**Affiliations:** 1 Oral Surgery Department, Faculty of Dental Medicine, Medical University-Plovdiv, 4000 Plovdiv, Bulgaria; ivan.chenchev@gmail.com; 2 Department of Prosthetic dental Medicine, Faculty of Dental Medicine, Medical University-Plovdiv, 4000 Plovdiv, Bulgaria; stefanzlatevdr@gmail.com; 3 Department of Department of Radiology, Dental and Physiotherapy Allergology, Faculty of Dental Medicine, Medical University-Plovdiv, 4000 Plovdiv, Bulgaria; dr.jordanov1956@abv.bg; 4 Head and Neck Maxillofacial Surgery, Department of Otoryngology, Tel-Aviv Sourasky Medical Center, Sackler Faculty of Medicine, Tel-Aviv University, Tel-Aviv 699350, Israel; mijiritsky@bezeqint.net

**Keywords:** alveolar ridge preservation, socket preservation, CBCT, bone resorption, intraoral scanner, CAD CAM, in vivo, virtual models

## Abstract

The aim of this study was to compare two different methods for evaluation of alveolar bone resorption after the socket preservation procedure. In the current study, 9 patients with a total of nine teeth indicated for extraction were included. Patients received alveolar ridge preservation with allograft (BoneAlbumin™, OrthoSera Dental, Gyor, Hungary) or Platelet-Rich fibrin (PRF). CBCT (Planmeca ProMax 3D, Helsinki, Finland), was taken at 1 week and 4 months after the socket preservation procedure. A 3D scan, obtained with Trios (3Shape, Copenhagen, Denmark) of the alveolar bone of the surgical site and the adjacent teeth at the place of extraction was performed during the surgical procedure, immediately after the graft placement in the alveolar socket, and after 4 months. Virtual study models were generated using the three-dimensional file processing software “Meshlab” (ISTI—CNR Rome Italy). The changes of alveolar height and width were measured and analyzed. Results were taken from both methods. Radiographic examination revealed that the average value of horizontal resorption is 0.6–2.4 mm, and vertical resorption is 0.46–2.8 mm. On virtual models, the average value for horizontal resorption is 1.92–3.64 mm, the vertical resorption value is 0.95–2.10 mm. The Trios intraoral scan can provide non-invasive and more accurate quantitative insights into the dimensional changes in the alveolar ridge after the bone remodeling process. More research is needed for verification of these results.

## 1. Introduction

Tooth extraction is one of the most common procedures in oral surgery. After tooth extraction, the alveolar ridge undergoes a series of biological and physiological events, which lead to resorption and remodeling of the bone. These changes are life-long and irreversible. The dimensional alterations are most pronounced during the first three months after tooth extraction [1,2]. Clinical studies demonstrate an average of 0.7–1.5 mm of vertical and 4.0–4.5 mm of horizontal bone resorption 6 months after tooth loss [3]. The healing process following tooth extraction results in more pronounced resorption on the buccal compared to the lingual/palatal aspect of the alveolar ridge [1,4].

Alveolar ridge preservation aims to reduce bone resorption and soft tissue collapse and to maintain the bone volume and density for the future dental implant placement. Studies reveal the beneficial effects of using different bone grafts and biomaterials in augmenting the socket following extractions as compared to the socket healing alone [5,6].

The amount of vertical and horizontal bone resorption has been investigated with a variety of methods. These range from clinical assessment with plastic cast models [1] or individually pre-fabricated acrylic stents [7], through radiographic evaluation [8], to histological studies in experimental animal models [4]. Despite the fact that the aforementioned methods are practiced and documented, they have several disadvantages: they are prone to errors due to technical and human limitations; they are time-consuming for the patient and the clinician; the patient is subjected to additional x-ray exposure; and most of them increase the treatment/research cost [9].

Intraoral scanners allow us to take accurate optical impressions of the dentogingival tissues and dental arches using only a beam of light [10]. The 3D surface models are a ‘virtual’ alternative to traditional plaster models [11]. Because of the high trueness and precision of the optical impressions intraoral scanners are widely used in prosthodontics, orthodontics and implant surgery. Studies on these features of intraoral scanners investigate them mainly on in vitro models [12,13,14]. Therefore, assessing virtual models of the bone obtained in vivo is more challenging, since there is the presence of factors such as blood and saliva in the mouth, reflection of light by intraoral structures, and operator or patient movement.

The aim of this study is to compare one of the well-established methods for evaluating bone resorption with CBCT (Planmeca ProMax 3D, Helsinki, Finland), and the usage of direct method to capture the patient’s bone directly with an intraoral scanning device and to produce a digital model that can be used to measure bone resorption rate before and after socket preservation procedure.

## 2. Materials and Methods

The Ethical Committee of Medical University, Plovdiv, Bulgaria, approved the present study (ethic code: P-2230/26.04.2018). Patients were enrolled after informed consent was obtained, and the protocol conformed to the ethical guidelines of the 1975 Declaration of Helsinki.

### 2.1. Patient Selection

Nine patients, with one extraction socket each, were included and treated in the oral surgery department of the faculty of dental medicine in Plovdiv, Bulgaria. All patients were consulted with the need for rehabilitation with oral implants. The inclusion criteria included: presence of tooth with indication for extraction, presence of adjacent teeth, ≥18 years of age, ASA (Physical Status Classification System, American Society of Anesthesiologist) I (normal healthy patient) or II (patient with mild systemic disease), good oral hygiene. The exclusion criteria included: ASA III or IV patients, uncontrolled diabetes, smokers (≥than 10 cigarettes/day), use of immunosuppressant medication, use of anticoagulants, adjacent tooth extractions, or a diffuse infectious process next to the site to be intervened.

### 2.2. Study Protocol

Two CBCTs were taken—one week after the tooth extraction and socket preservation procedure, and four months after. After elevating the mucoperiosteal flap the alveolar bone was scanned with Trios intraoral scan (Trios 3Shape, Copenhagen, Denmark) and virtual models were generated. Four months after the extraction, the same scans were taken again.

### 2.3. Surgical Technique

After administering adequate local anesthesia, an intrasulcular incision around the tooth to be extracted was followed by two vertical diverging incisions reaching the mesial/distal papilla of the adjacent teeth, and a mucoperiosteal flap was elevated both from the buccal and lingual/palatal site. The tooth was extracted as atraumatically as possible. The socket was carefully debrided and filled with bone graft material (Platelet-Rich Fibrin—PRF or BoneAlbumin™, OrthoSera Dental, Gyor, Hungary) The site was then scanned with a Trios intraoral scanner (Trios 3Shape, Copenhagen, Denmark) and a three-dimensional virtual model of the bone walls around the socket was obtained. Following a periosteal slitting, the mucoperiosteal flap was adapted without tension in order to obtain a primary closure of the socket, and it was sutured with 0000 non-resorbable thread.

Four months after the socket preservation procedure, a surgical re-entry for implant placement was performed. After elevating a mucoperiosteal flap, before implant placement, the site was scanned with Trios intraoral scan, and a three-dimensional virtual model of the healed bone was obtained.

### 2.4. Postoperative Care

Postoperatively, patients were prescribed non-steroid anti-inflammatory drugs for three days and 0.12% chlorhexidine rinse twice a day for 2 weeks. After 10 days, sutures were removed.

### 2.5. Radiographic Examination on CBCTs

**Figure 1:**
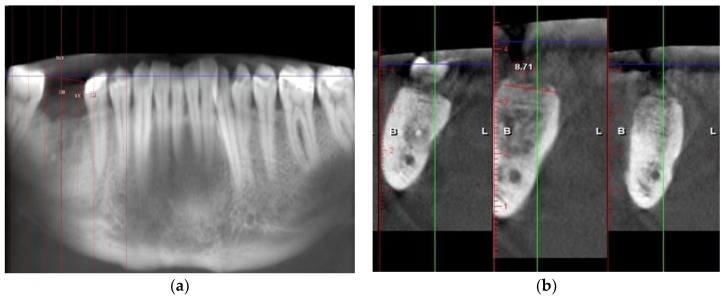
Radiographic examination and measurements in (**a**) vertical and (**b**) horizontal dimension.

Two consecutive CBCTs were obtained—one immediately after the socket preservation procedure and one 4 months after. All of the CBCT scans were performed at the faculty of dental medicine in Plovdiv. Horizontal and vertical resorption were measured using the measurement tool “measure length” in the toolbox (Planmeca Romexis Viewer 4.4.3, Planmeca, Helsinki, Finland). To measure the vertical resorption, a horizontal line was made between the occlusal surfaces of the adjacent teeth, and to assess the vertical resorption, a horizontal line between the occlusal surfaces of the adjacent teeth was made. This served as a reference for constructing perpendicular lines towards the bone. Measurements were taken at three points on each model—before and after 4 months of healing (Figure 1). The results were obtained by calculating the difference between both of the CBCTs—before and 4 months after (Table 1).

### 2.6. Three-Dimensional Virtual Models Evaluation

**Figure 2:**
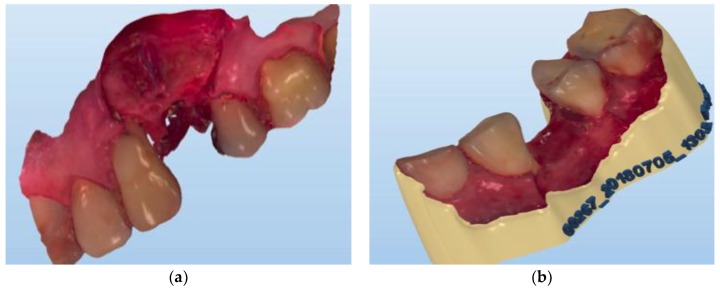
Virtual models of the operative field—bone dimensions during socket preservation (**a**) and 4 months after before implant placement (**b**).

The resulting three-dimensional replications of the operating field during socket preservation and four months after are in “.stl” format (Figure 2).

**Figure 3:**
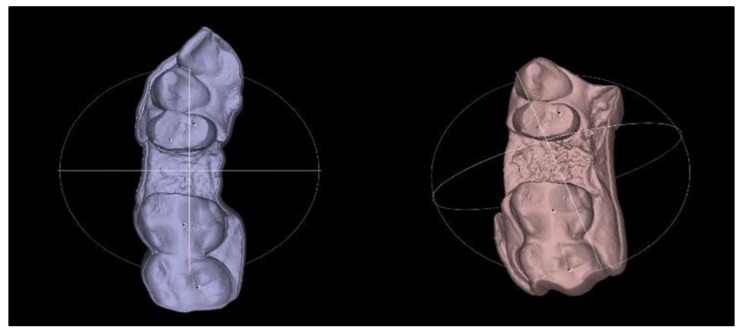
Process of superimposition of two models—before and after. Selecting four different points on the adjacent teeth to create alignment of the virtual models.

**Figure 4:**
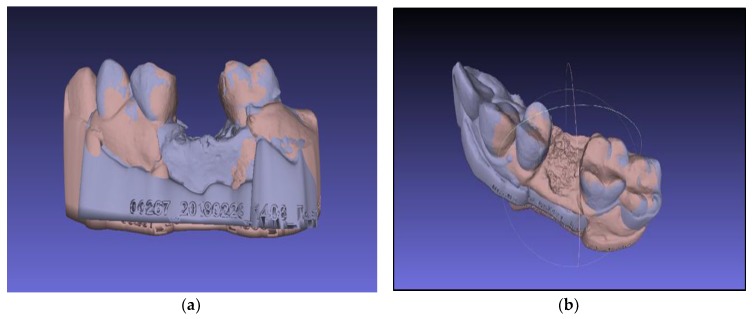
Superimposed STL models. (**a**) buccal aspect of socket preservation with PRF; (**b**) occlusal aspect of socket preservation with BoneAlbumin.

The following is a procedure for importing the models into the three-dimensional file processing software, “Meshlab” (ISTI—CNR Rome Italy), through the submenu of the program—“File> import”. The models are superimposed on each other in the three-dimensional space with the function “Align” (Figure 3 and Figure 4).

The alignment is performed in the following order:

- Selection of the base model—choose a model that is tightly oriented at the base of the work area defined by the axes “x” and “y”
- Fix the selected model using the “Glue mesh here” command
- Equation of points with the point-based gluing function
- Selection of the “processing” function, by which the models are finely tuned by the “ICP—iterative closest point” method

The models are aligned to each other based on the surfaces that have not undergone alterations during the post-operative period—i.e., the adjacent teeth.

**Figure 5:**
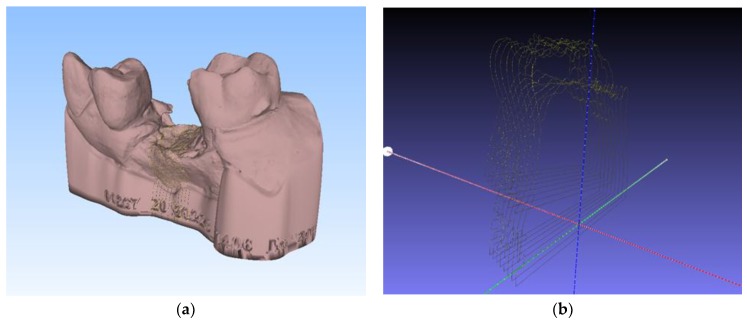
Planar sectioning of the models. (**a**) Constructed planar sections on the superimposed models; (**b**) Constructed planar sections without the model.

The area of interest is the surgical site where two surfaces are obtained immediately after extraction and four months post-operative. To evaluate bone resorption, it is necessary to linearly measure the distance between the aligned virtual models in different areas of the defect. The latter is done using the “Compute planar section” function in the menu—“Filters > Geometric measurements and computations” (Figure 5).

**Figure 6:**
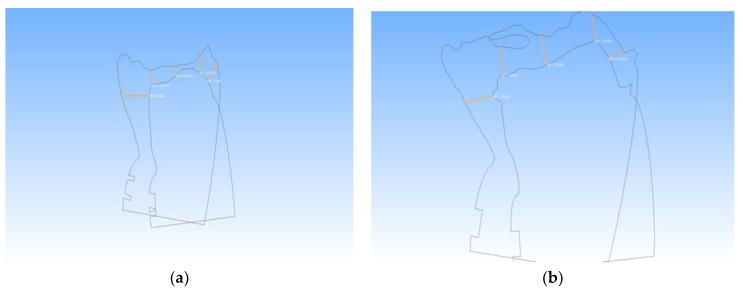
Measuring the line spacing between the two outer contours of the models in different slices. (slice −1.5 and 2.5). (**a**) slice 1.5 mm and (**b**) slice 2.5 mm.

To obtain information for all areas of the defect, multiple cuts are made at 0.5 mm. The number of cutting planes/slices depends on the size of the extraction site (Figure 6). Each of the resulting slices indicates the outline (outer edges) of one of the two .stl models—immediately after extraction or 4 months post-operatively. Since the models were aligned in the previous step, the set position of both cuts coincides with the vertical axis of orientation, i.e., the layout of the contours is strictly the same. Each of the slices is retained in the program as a separate “grid”, with a name indicating its position relative to the three planes of space.

The “measure tool” was used to measure the line spacing between the two outer contours of the models (Figure 6). For each pair of slices, the following measurements were performed: two horizontal distances are measured—vestibular and lingual; and three vertical distances—vestibular, lingual and in the central area of the defect. All measurements are stored in text format and as screenshots of the program.

## 3. Results

### 3.1. Radiographic Analysis

As a result of the measurements performed on the CBCTs made up to 1 week after the socket preservation procedure and 4 months after the manipulation, the lowest average value of the horizontal resorption was 0.6 mm and the highest was 2.4 mm. The lowest measured vertical resorption is 0.46 mm and the highest was 2.8 mm.

### 3.2. Virtual Models Analysis

**Figure 7:**
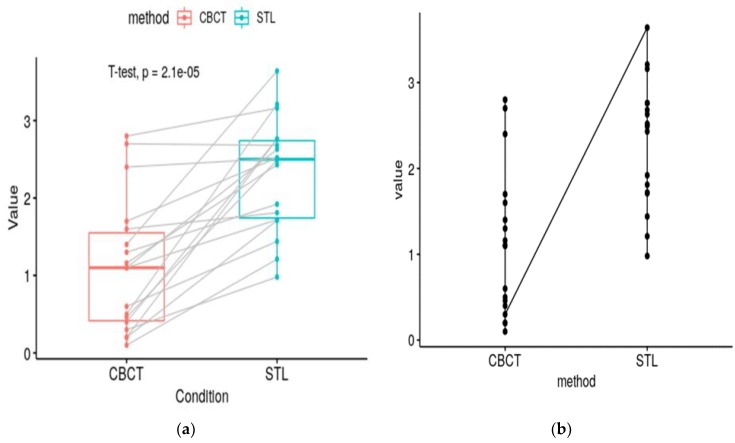
Graphical representation of the result. (**a**) A boxplot with pairwise comparison and a *t*-test statistic; (**b**) line plot of the results.

To assess the horizontal resorption occurring for each cut, the vestibular and lingual distances were calculated. An arithmetic mean was calculated, which indicated that the central trend of bone loss was in the horizontal direction. To assess the vertical resorption occurring for each of the slices, the arithmetic mean of the three linear measurements was calculated. The mean value obtained from all slices showed a central trend of bone resorption in the vertical direction. Mean values were calculated for all slices at all three measurement points, allowing for the assessment of the resorption process in the different zones of the preserved area. A *t*-test was used to compare the linear changes in the different areas of the defect. Visually, the results are represented by “boxplot” graphs (Figure 7).

As a result of the performed manipulations, 740 measurements were made, including 296 for horizontal resorption and 444 for vertical resorption. The smallest number of slots for a pair of models was 8 and the largest was 21. The lowest average value for horizontal resorption was 1.92 mm, and the highest was 3.64 mm. The lowest measured vertical resorption value was 0.95 mm, and the highest was 2.10 mm.

## 4. Discussion

The aim of this study was to compare the bone resorption linear measurements of the height and width of 9 preserved alveolar sockets, obtained with two different methods. A novel 3D analysis allowed the evaluation of dimensional alterations of the bone following extraction and socket preservation procedures in human patients. In the present study, this method is compared to a well-established radiographical method for bone resorption assessment—CBCT. To assess vertical and horizontal resorption, authors have suggested different methods, ranging from clinical assessment with plastic cast models [1], through individually pre-fabricated acrylic stents [7], to radiographic evaluation [8,15], and histological studies in experimental animal models [4].

Researchers rely on two main evaluation techniques—clinical and radiographical. Although these are well-established, they have several drawbacks. Patients are subjected to additional X-ray exposure, and clinical methods are more time-consuming and are prone to more measurement errors [16,17]. The use of various radiographic methods, including CBCT for the assessment of post-extraction resorption of the alveolar bone, is used by many authors [18,19,20]. The clinical findings from the CBCT in our pilot study are comparable to the results from socket preservation therapies presented in the literature [19,21]. According to the review article by Van der Wejden et al. [22] on bone resorption in post-extraction sockets, the clinical loss in width (3.87 mm) is greater than the loss in height, as assessed both clinically (1.67–2.03 mm) and radiographically (1.53 mm). Chappuis et al. [23] introduced a novel 3D method utilizing digital model superimpositions based on two consecutive CBCTs in order to characterize the extent of bone loss and to identify risk zones and the respective modulating factors for facial bone resorption. They report 3.5 times more severe bone loss in vertical dimension compared to other experimental pre-clinical studies. In another study, by Gultekin et al. [24], bone volumetric changes after GBR and autogenous ramus block bone grafting were evaluated through CBCT. Although well established, this method has disadvantages. The wide variety of image quality and exposure rate require optimization. The accuracy of the linear measures on CBCT (200 μm) can sometimes reach fivefold inaccuracy levels [25].

One of the main advantages of using intraoral digital impressions instead of the conventional ones is the reduction of the cost of the materials and the increased patient comfort [26]. We have not found data in the literature regarding the use of 3D intraoral scan for measuring resorption of bone following preservation of the alveolar ridge. The concept of using 3D intraoral scan for this purpose is based on the ability to obtain very accurate 3D virtual models (in vivo bone structures) with unparalleled accuracy—below 20 micrometers—and precision—below 10 micrometers [27]. According to the study by Mangano et al. [14], Trios^®^ showed a trueness of 71.2 μm and a precision of 51.0 μm. In another study [13], Trios^®^ showed 50.2 ± 2.5 μm trueness and 24.5  ±  3.7 μm precision. Processing the resulting 3D virtual models makes possible precise measurements in horizontal and vertical dimensions, and simple and precise calculation of differences in the measured sizes and volumes. In a previous study of ours [9], we proposed 3D intraoral scan to assess the resorption of the bone in phantom models. The results showed potential for using this technique in clinical settings.

Several studies have investigated the application of intraoral scanners and virtual models in implantology, and particularly for the investigation and assessment of dimensional alterations of facial soft tissue morphology in the esthetic zone [28] and the evaluation of the stability of peri-implant soft tissues over time [29]. Yet we could not find any studies in the literature supporting the use of intraoral scanners for obtaining changes in bone morphology in vivo after socket preservation procedures. The proposed method has limitations in cases where adequate conditions for optical impressions cannot be achieved, such as where there is inadequate dryness (e.g., blood, saliva, exudation cannot be controlled), or the inability to ensure direct line of sight between the scanner’s tip and the surgical site. These can decrease the quality of the virtual model or prohibit the acquisition of important details, rendering the digital impression unusable.

## 5. Conclusions

The results obtained with the CBCTs and the virtual models reveal a significant difference. Radiographic analysis is a well-established method for measuring the bone resorption pattern after augmentation procedures, but the patient is subjected to additional X-ray exposure. On the other hand, virtual models obtained with the Trios intraoral scan can provide non-invasive and more accurate quantitative insights into the dimensional changes in the alveolar ridge after the bone remodeling process. More research is needed for verification of these results.

## Figures and Tables

**Table 1:** Mean values for horizontal and vertical bone resorption measured on the CBCT scans and virtual models.

Method	CBCT		Virtual Model (.stl)	
Direction	Horizontal (mm)	Vertical (mm)	Horizontal (mm)	Vertical (mm)
Case Number				
1	0.8	1.4	3.64	2.10
2	0.6	1.6	2.63	1.15
3	1.1	1.1	2.28	1.84
4	0.1	1.16	2.43	0.805
5	0.8	2.7	2.68	1.84
6	0.8	0.46	2.49	1.14
7	1.2	0.5	1.92	0.95
8	2.4	2.8	3.64	1.68
9	0.3	1.7	2.63	0.657
